# KIT-Mutant Melanoma: Understanding the Pathway to Personalized Therapy

**DOI:** 10.3390/cancers17223644

**Published:** 2025-11-13

**Authors:** Aditi Kaveti, Ryan J. Sullivan, Hensin Tsao

**Affiliations:** 1 Carle Illinois College of Medicine, Champaign, IL 61801, USA; akaveti2@illinois.edu; 2 Massachusetts General Hospital Cancer Center, Boston, MA 02114, USA; rsullivan7@mgh.harvard.edu; 3 Department of Dermatology, Massachusetts General Hospital, Harvard Medical School, Boston, MA 02114, USA

**Keywords:** KIT, melanoma, mutation, exon, inhibitor, targeted therapy, immunotherapy

## Abstract

Melanoma is a highly heterogeneous disease, with unique genetic subtypes that influence clinical behavior and treatment response. While targeted therapies and immunotherapy have transformed care for more common types of melanoma, optimal strategies for *KIT*-mutant melanoma are less well-defined. In this review, we summarize the associations between *KIT* mutations and specific clinico-pathologic patterns, including their enrichment in acral, mucosal, and chronically sun-damaged melanomas. We detail the spectrum of *KIT* mutations across relevant exons, noting how mutation subtype influences sensitivity to targeted therapies. We will then analyze several therapeutic trials and case reports that describe the use of c-KIT inhibitors as well as immune checkpoint inhibitors in melanoma treatment. We also discuss early findings supporting combination strategies that may enhance therapeutic outcomes. Finally, we identify critical gaps in understanding the mechanisms of resistance, CNS progression, and the immunogenic landscape of *KIT*-driven melanoma. Deeper insight into the function of KIT and its interaction with therapeutic pathways is essential to optimizing treatment sequencing and tailoring personalized strategies that improve outcomes in this patient population.

## Simple Summary

Skin cancer encompasses a range of diseases that differ widely in their genetic drivers, clinical behavior, and treatment response. Among these, melanoma is the most aggressive form and includes several uncommon subtypes, such as acral, mucosal, and chronically sun-damaged melanomas, that often contain activating mutations in the *KIT* gene. These tumors behave differently from other forms of melanoma and frequently demonstrate variable responses to standard targeted or immune-based therapies. The purpose of this review is to synthesize and interpret current evidence on how specific *KIT* gene mutations shape clinical outcomes, therapeutic sensitivity, and resistance mechanisms. We highlight findings from key clinical studies evaluating targeted tyrosine kinase inhibitors and immune checkpoint inhibitors, emphasizing the most responsive mutation sites, comparative efficacy across agents, and treatment sequencing considerations. The review also explores clinical implications and highlights critical unanswered questions in this evolving field. Together, these insights provide a practical framework to guide ongoing research and inform more effective, personalized therapeutic strategies for patients with *KIT*-mutant melanoma.

## 1. Introduction

Melanoma is the most aggressive form of skin cancer and accounts for the vast majority of skin cancer-related deaths despite comprising a smaller proportion of skin cancer cases overall [1]. Although early stage melanoma is often cured with surgical resection, advanced disease carries a high risk of metastasis and poor survival outcomes [2]. The biological heterogeneity of melanoma is driven by diverse genetic alterations that inform clinical behavior, prognosis, and therapeutic response [3]. Most cutaneous melanomas harbor activating mutations in *BRAF* or *NRAS*, but a distinct subset is defined by the oncogenic activation of *KIT* through somatic mutations [4].

The receptor tyrosine kinase KIT (also known as CD117), encoded by the *KIT* proto-oncogene on chromosome 4q11-12, plays key roles in cell survival, proliferation, and differentiation via activation by its ligand stem cell factor [5,6,7]. *KIT* mutations are most frequently observed in acral, mucosal, and chronically sun-damaged (CSD) melanomas and are rarely found in non-CSD cutaneous melanoma [7]. The mutational spectrum of *KIT* in melanoma includes alterations across several exons, with classic activating mutations in particular that predict sensitivity to KIT inhibitors in select patients.

KIT is also a well-established therapeutic target in gastrointestinal stromal tumors (GIST), where activating mutations drive disease pathogenesis and predict robust responses to tyrosine kinase inhibitors (TKI) such as imatinib [8,9]. However, the therapeutic landscape in *KIT*-mutant melanoma has proven more complex, with variable response rates to TKIs and emerging data on the potential role of immune checkpoint inhibitors (ICI).

In this review, we summarize the current understanding of *KIT*-mutant melanoma, beginning with its clinico-genetic features and the spectrum of *KIT* mutations implicated in diseases. We then review the therapeutic landscape, including the role of targeted therapies and ICIs, and conclude by outlining unanswered questions, including ongoing challenges, mechanisms of resistance, and areas for future research.

## 2. Methods

We conducted a structured literature search in PubMed to identify studies published between 2000 and 2025 related to *KIT*-mutant melanoma. The search terms included “KIT”, “mutation”, “melanoma”, “tyrosine kinase inhibitor”, and “immunotherapy.” Reference lists of relevant reviews and clinical studies were also screened to capture additional publications.

Eligible articles included clinical trials, cohort studies, case series, and case reports describing patients with confirmed *KIT*-mutant melanoma, including acral, mucosal, and CSD subtypes. Studies that reported both *KIT* mutation and amplification were included for completeness, but outcomes were analyzed and interpreted primarily in the context of mutation-positive disease. Reports limited to amplification without confirmed mutation and studies focused on non-melanoma malignancies were excluded.

## 3. Clinico-Genetic Correlations

*KIT*-mutant melanoma constitutes a rare but well-defined clinico-genetic subset characterized by distinct histological subtypes, anatomic locations, and patient demographics. Although present in only about 3% of all melanomas, *KIT* mutations are disproportionately found in tumors arising from acral sites, mucosal surfaces, and chronically sun-damaged skin [7].

Patient characteristics also contribute to the clinico-genetic profile of *KIT*-mutant melanoma. These tumors are diagnosed more frequently in older people, particularly those 60 years or older, and tend to occur without strong associations with sex, Breslow thickness, ulceration, or stage [7,10]. The presence of a *KIT* mutation has, however, been associated with shorter recurrence-free survival and distant metastasis-free survival [11]. Additionally, racial differences have been observed in subtype-specific mutation patterns. In one meta-analysis, *KIT* mutations were significantly associated with mucosal melanoma in white patients and acral melanoma in Asian patients [7,10]. These findings reflect both environmental exposure patterns and the prevalence of different melanoma subtypes between populations.

**Figure 1:**
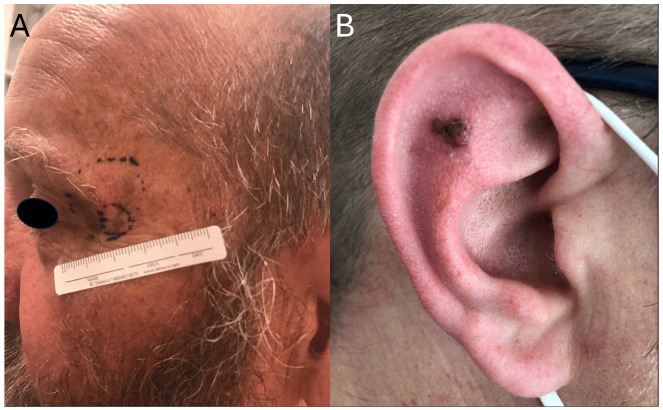
Clinical presentation of *KIT*-mutant melanoma with involvement of sun-exposed areas. (**A**) Periocular lesion with biopsy site marked. (**B**) Pigmented lesion of the auricle.

In terms of anatomical distribution, *KIT* mutations are enriched in melanomas that arise in mucosal tissues (e.g., oral, genital, and anal surfaces) and acral locations (e.g., palms, soles, and subungual areas). They have also been reported on skin with CSD, such as the head and neck and non-acral extremities (Figure 1). In contrast, they are rarely found in tumors located on the trunk [7,10]. This anatomic distribution further supports the hypothesis that *KIT*-driven melanomas are biologically and clinically distinct from other melanoma subtypes that are primarily driven by more common alterations involving the MAPK pathway.

## 4. Spectrum of *KIT* Mutations in Melanoma

**Figure 2:**
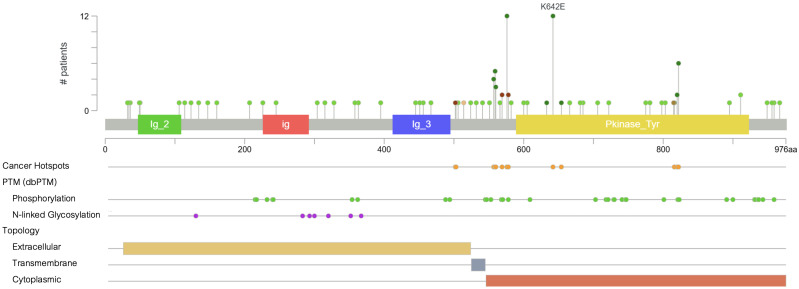
Distribution of *KIT* mutations across functional domains. Green markers represent missense mutations, which constitute the majority of mutations observed in melanoma.

The molecular spectrum of *KIT* mutations in melanoma is defined by a set of recurrent activating alterations that cluster within specific domains of the KIT receptor tyrosine kinase. These mutations are most commonly point mutations, specifically missense substitutions, which drive constitutive signaling through ligand-independent activation [7] (Figure 2). Population-level studies estimate that approximately 11–23% of acral melanomas, 15–21% of mucosal melanomas, and up to 27% of CSD melanomas harbor *KIT* mutations, whereas the prevalence in non-CSD cutaneous melanoma is generally less than 5% [7,10,12,13]. While *KIT* amplification can occur, it is less frequently observed and typically does not co-occur with activating mutations, suggesting that amplification alone may be insufficient to predict therapeutic response [13]. Notably, *KIT* mutations are usually mutually exclusive of *BRAF* and *NRAS* mutations, which are the most common oncogenic drivers in cutaneous melanoma [7].

*KIT* mutations in melanoma are most commonly localized to exons 11, 13, and 17, which encode the juxtamembrane and kinase domains of the receptor [7,14,15]. Exon 11 is the most frequently affected, accounting for approximately 50–60% of cases, and includes the L576P mutation, which alone represents about 30% of all reported *KIT* mutations. Other recurrent substitutions in exon 11 include V559A and D579H and deletions affecting W557/K558, all of which disrupt the auto-inhibitory function of the juxtamembrane segment and lead to constitutive activation of the kinase [16].

Exon 13, part of the kinase domain I, harbors the K642E mutation, observed in 10 to 15% of *KIT*-mutant melanoma cases [16]. Preclinical murine models of K641E in GISTs have demonstrated that this mutation alone is sufficient to initiate tumorigenesis. The corresponding human variant, K642E, differs only by residue numbering due to species-specific variation in the KIT protein sequence. In melanocytes, it can also drive hyperproliferation and progression to invasive melanoma, mirroring features of human mucosal melanoma [17,18]. This glutamic acid substitution works by enhancing catalytic activity and has been associated with clinical sensitivity to imatinib and other tyrosine kinase inhibitors [7,16].

Mutations that occur in exon 17 include the D820Y mutation and others that comprise roughly 5–10% of the mutational burden. Though rarer, they have been reported in acral melanoma and have shown variable treatment responses, including resistance to imatinib and potential sensitivity to sorafenib, a multikinase inhibitor [19].

Together, L576P and K642E account for over half of all *KIT* mutations in melanoma and have become prototypical examples of “hotspot” mutations [16]. These mutations are both biologically significant and clinically actionable, conferring sensitivity to KIT inhibitors like imatinib, sunitinib, and nilotinib [7]. As a result, they are frequently prioritized in molecular testing panels, particularly in patients with acral or mucosal melanomas where *KIT* alterations are more prevalent. In settings where genetic testing is unavailable, empirical treatment with KIT inhibitors may still be considered for patients with high clinical suspicion based on subtype and site of disease.

Outside of these hotspots, a long tail of rare and novel mutations has been documented, often in isolated case reports [7]. For example, gain-of-function mutations such as S628N in exon 13 have been identified and shown to respond to KIT inhibition in vitro [20]. This growing list of mutations illustrates the genetic heterogeneity of *KIT*-mutant melanoma and underscores the utility of comprehensive sequencing, particularly in cases with unclear clinical features or resistance to standard therapies.

While the prevalence of *KIT* mutations remains low in the overall melanoma population, their consistent enrichment in specific histologic subtypes and well-defined molecular mechanisms of action have made them an important biomarker subgroup. Identifying not only the presence of *KIT* alterations, but also their specific exon location and amino acid change, is crucial for guiding therapeutic decision-making and predicting drug response. Preclinical studies further illustrate that specific mutations, such as K641E, can directly contribute to aggressive disease phenotypes, underscoring the biological impact of precise mutation profiles. As our understanding of these mutations evolves, so does the potential to tailor targeted therapies to a broader range of patients with *KIT*-driven disease.

## 5. Targeted Therapies

An early landmark study by Curtin et al. identified *KIT* genetic aberrations in acral, mucosal, and CSD melanomas, which prompted the evaluation of several small-molecule inhibitors that were already used for GISTs, at least 80% of which harbor a *KIT* mutation [12]. Today, the agents with the most data are imatinib, nilotinib, dasatinib, and sunitinib, while newer compounds such as regorafenib and ripretinib have entered early clinical trials. Across these studies, therapeutic benefit has consistently correlated with the presence of activating *KIT* mutations, particularly L576P and K642E. These activating variants serve as the clearest biomarkers of drug sensitivity, whereas amplification alone has shown little to no predictive value for response.

### 5.1. Imatinib

Imatinib, a first-generation TKI, has demonstrated meaningful clinical activity against multiple tyrosine kinases such as c-KIT, CSF1R, ABL, FLT3, and PDGFR. Multiple phase II trials were conducted in the early 2000–2010s, enrolling patients whose melanomas harbored activating *KIT* mutations or amplifications in acral, mucosal, or CSD melanoma [21,22,23,24,25,26]. Objective response rates (ORR) ranged from 15% to 29%, and disease control rates (DCR) ranged from 36% to 60%. Median progression-free survival (PFS) was generally short, between 2.8 and 4.2 months, and overall survival (OS) ranged from 10.7 to 13.1 months (Table 1). The strongest predictors of benefit were the presence of specific *KIT* point mutations, particularly in exon 11 (e.g., L576P) and exon 13 (e.g., K642E), rather than *KIT* amplification alone, which conferred poor outcomes [22]. In Hodi et al., patients with *KIT* mutations had a 77% DCR versus 18% in those with *KIT* amplification only [24]. These results emphasize that therapeutic benefit is driven by activating mutations such as L576P and K642E, whereas amplification-only cohorts perform worse than mutation-positive cases. Extrapolating TKI efficacy to amplification-only disease risks overstating potential benefit and should be avoided.

In one multicenter phase II trial, high-dose imatinib demonstrated no clinical efficacy and significant toxicity in an unselected population of metastatic melanoma patients. This outcome highlights the critical importance of molecularly defining a population most likely to benefit from KIT inhibition [40].

Case reports provide valuable insight into how specific *KIT* mutations influence clinical response to imatinib. In Beaudoux et al., a patient with metastatic melanoma harboring an L576P (exon 11) and T847M (exon 18) double mutation demonstrated a rapid and near-complete metabolic remission following imatinib, with resolution of all visible metastases except for a small pleural nodule [41]. Although lung and pleural metastases eventually recurred at six months, the patient responded to subsequent therapies and remained alive with minimal residual disease 18 months after starting imatinib [41]. In Brown et al., a patient with a K642E mutation (exon 13) experienced a complete response (CR) lasting over 18 months and remained in remission at the time of publication [42].

Conversely, patients with less common mutations have shown more variable responses. A case involving a rare T632I mutation reported disease progression despite initiating imatinib based on its proximity to K642E in the kinase domain [43]. Similarly, a patient with a C443S mutation (exon 8) reported marked symptomatic and radiographic improvement with imatinib but was ultimately followed by progressive systemic metastases at week 15 and death shortly thereafter [44]. Notably, this report reinforces imatinib’s limited central nervous system penetration, as intracranial relapse can occur even when extracranial disease is initially controlled [45]. Collectively, these studies highlight the predictive value of mutational profiling, particularly for known activating mutations, though resistance to imatinib remains a major limitation and underscores the need for more durable therapeutic strategies.

### 5.2. Nilotinib

Nilotinib is a second-generation KIT/ABL TKI developed as a more potent alternative to imatinib. While initially used in chronic myeloid leukemia, it has also been evaluated in *KIT*-driven melanomas, particularly acral and mucosal subtypes [46]. The TEAM trial, a phase II study, treated 42 patients with *KIT*-mutant metastatic melanoma using nilotinib 400 mg BID. The study reported an ORR of 26.2%, a DCR of 74%, a median PFS of 4.2 months, and a median OS of 18.0 months [27]. Most responders had exon 11 mutations, especially L576P, highlighting the relevance of juxtamembrane domain alterations. Nilotinib was less effective in tumors harboring mutations in other exons.

The prospective NICAM phase II trial further evaluated nilotinib in *KIT*-mutant acral and mucosal melanoma. Among 26 evaluable patients, objective responses at 12 weeks were observed in 16% (3/19) of those with exon 11 mutations, 25% (1/4) with exon 13, and 67% (2/3) with exon 17, although the small sample sizes limited interpretation and no significant differences were detected across exon groups. Median PFS was 3.7 months, consistent with prior reports, while median OS was 7.7 months [28]. No correlation was found between *KIT* copy number amplification and treatment outcomes, reinforcing that nilotinib benefit primarily occurs in tumors with activating *KIT* mutations rather than amplification alone.

Several additional phase II studies support these findings, though with slightly varying efficacy. Across these trials, ORRs ranged from 16% to 23%, with DCRs between 23% and 78% [29,30,31,32]. One French multicenter trial reported a best ORR of 16% and a DCR of 64% [29]. Responders in this study also showed a marked drop in *KIT* mutation burden during treatment. Cho et al. demonstrated durable responses in patients with both *KIT* mutations and high-level *KIT* amplification (copy numbers > 240), with one patient experiencing a 10+ month response despite no detectable mutation [30]. Meanwhile, the study by Carvajal et al. in patients with prior imatinib exposure found an ORR of 16% at 12 weeks and DCR of 52.6% [31].

Nilotinib has also shown clinical benefit in select cases. One patient with metastatic vaginal melanoma, lacking detectable *KIT* mutations or amplification across exons 9, 11, 13, 17, and 18, achieved a durable CR lasting nearly five years [47]. This case suggests the possibility of undetected *KIT* alterations or alternative mechanisms of nilotinib sensitivity in mucosal melanoma.

While nilotinib has a favorable oral dosing regimen and a tolerable toxicity profile, primarily characterized by fatigue, rash, and transaminitis, its efficacy varies by *KIT* mutation subtype. It has been shown to be most effective in tumors harboring *KIT* exon 11 mutations but may offer minimal benefit in amplified and non-mutant tumors as well. However, like imatinib, its clinical utility appears limited to a subset of biologically responsive melanomas.

### 5.3. Dasatinib

Dasatinib is a multitarget TKI with activity against KIT, BCR-ABL, PDGFR, and EPHA2 and several SRC family kinases (c-SRC, YES, LCK, and FYN) [48]. Its potential role in *KIT*-mutant melanoma was explored in early preclinical work showing that the L576P substitution in exon 11 conferred greater potency for dasatinib over imatinib [49]. This was further expanded by Woodman et al., who demonstrated that the L576P substitution in exon 11 shifts KIT toward active conformation, reducing imatinib binding affinity but preserving dasatinib sensitivity [50]. This hypothesis was further supported by in vitro data and the treatment of two patients with metastatic melanoma harboring L576P mutations. Both had marked tumor regressions (>50% metabolic response by PET) despite one having previously failed imatinib.

Two multicenter phase II trials have since evaluated dasatinib in advanced melanoma. In patients with *KIT*-mutant acral, mucosal, or vulvovaginal melanoma, dasatinib was administered at 70 mg twice daily [33]. Among 22 patients treated with 70 mg twice daily, 4 had a partial response (PR, 18.2%) and 7 had stable disease (31.8%) [33]. Responses were short-lived however, with a median PFS duration of 2.7 months among *KIT*-mutant patients. Importantly, none of the seven patients harboring the L576P mutation achieved a PR.

A second phase II trial focused on patients with stage III/IV chemotherapy-naive unresectable melanoma. Across 36 evaluable patients, toxicities such as fatigue, dyspnea, pleural effusions, and gastrointestinal symptoms were common, prompting a reduction in dasatinib from 100 mg twice daily to 70 mg twice daily [34]. The ORR was low at 5%, as only two had confirmed PRs lasting 64 and 24 weeks. One of these responders harbored a *KIT* K642E exon 13 mutation. The remaining responder had wild-type *KIT*, suggesting possible *KIT*-independent effects in a small subset of patients. Notably, an exon 11 deletion mutation was identified in one non-responder, underscoring the heterogeneity of clinical responses even among tumors with presumed sensitizing mutations.

While dasatinib shows activity in *KIT*-mutant melanoma, especially in preclinical models and isolated cases, its clinical benefit is constrained by modest efficacy, short duration of response, and a narrow therapeutic window due to toxicity. Consequently, it is not considered a preferred front-line option but may be considered in select patients, particularly those with known imatinib-resistant mutations for which preclinical data suggest greater dasatinib sensitivity.

### 5.4. Other KIT/Multikinase Inhibitors

Sunitinib is a multikinase inhibitor that targets KIT, VEGFR, PDGFR, among others, and has been evaluated in multiple phase II studies for *KIT*-altered melanoma. Across trials, efficacy has been highly variable, with ORRs ranging from 8% to 40% [35,36,37]. In a larger cohort, including both *KIT*-mutant and wild-type tumors, responses were modest (4/52 PRs, 8%) and not clearly associated with *KIT* status (3 PRs in *KIT* WT, 1 PR in *KIT*+). At the same time, a smaller study limited to *KIT*-mutant patients demonstrated higher response rates (1/12 CR 8.3%; 3/12 PR 25%), particularly in those with exon 11 mutations [35,36]. More recently, in patients with diverse *KIT*-mutated tumors, sunitinib produced PRs in squamous cell carcinoma but no confirmed responses in the melanoma subset, reinforcing the drug’s variable efficacy [37].

Other broad-spectrum TKIs with anti-KIT activity have produced isolated but instructive signals. Sorafenib (RAF/KIT/VEGFR/PDGFR inhibitor) suppressed tumor growth in KIT-over-expressing melanoma models, and a complete but transient (five-month) response has been documented in a patient with anal mucosal melanoma harboring the exon 11 p.V560D alteration [51,52]. Masitinib, which shares activity against KIT, PDGFR, and LYN, induced rapid regression, including central nervous system metastases, within four weeks in a *KIT* exon 11 mutant esophageal melanoma [53].

Newer agents originally developed for GISTs are now being investigated in melanoma. Regorafenib, which targets KIT and VEGFR pathways, achieved an ORR of 30.4% and median PFS of 7.1 months in a 23-patient phase II study [38]. Responses were seen across mutation sites, including exons 11, 13, and 17, and in patients previously treated with immune checkpoint inhibitors. Ripretinib, a type II switch-control TKI, showed a confirmed ORR of 23% (1 CR, 5 PRs) and a median PFS of 7.3 months in a phase I expansion cohort of 26 patients [39]. Activity was greatest among patients with *KIT* exon 11 mutations (ORR 44%), and the drug was well tolerated.

Together, these data suggest that broader-spectrum TKIs may provide benefit in select patients with *KIT*-altered melanoma, particularly those with resistance to first-line agents, though further investigation in larger, molecularly stratified cohorts is warranted.

## 6. Immunotherapy in *KIT*-Mutant Melanoma

**Figure 3:**
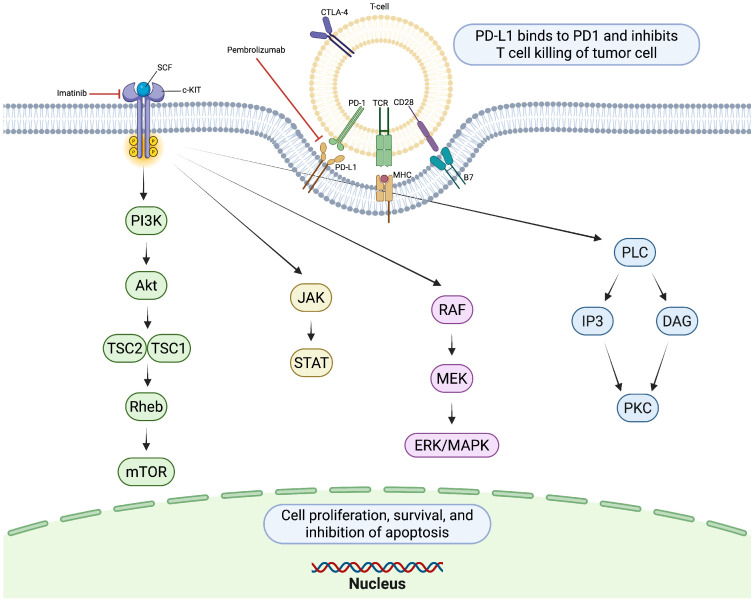
KIT receptor activation by stem cell factor (SCF) triggers dimerization and autophosphorylation, initiating downstream signaling cascades. PD-1/PD-L1 signaling suppresses T-cell activity, limiting antitumor immunity. Created in https://BioRender.com.

Immune checkpoint inhibitors, particularly anti-PD-1 and anti-CTLA-4 agents, have become the cornerstone of treatment in advanced melanoma. In cutaneous melanoma, most commonly driven by BRAF or NRAS mutations, ICIs have demonstrated substantial improvements in overall survival and durable clinical responses [54]. The success of therapies such as nivolumab, pembrolizumab, and ipilimumab has established immunotherapy as a foundational treatment strategy in this population (Figure 3).

In contrast, *KIT* mutations are relatively uncommon, occurring in only 1–3% of cutaneous melanomas and more frequently in mucosal, acral, and chronically sun-damaged subtypes [7]. Given the rarity of *KIT*-driven melanoma and its distinct molecular features—including a lower tumor mutational burden and non-UV-driven pathogenesis—the efficacy of ICIs in this subset remains less well characterized.

In a retrospective study from MD Anderson, 55 patients with advanced *KIT*-mutant melanoma were treated with anti-CTLA-4 and/or anti-PD-1 therapy. Among 35 patients who received anti-CTLA4 therapy, the ORR was approximately 20%, with median OS and PFS of 11.8 and 3 months, respectively [55]. Twenty patients treated with anti-PD-1 therapy demonstrated a slightly higher ORR of 35%. Patients treated with both anti-CTLA-4 and anti-PD-1 showed limited dual benefit: only one responded to both agents and seven responded to just one [55]. Clinical response to at least one ICI was observed across *KIT* exon mutations 2, 11, 13, and 17, but there was no clear correlation between *KIT* mutation subtype and clinical benefit, suggesting that immune checkpoint inhibition may be independent of *KIT* exon mutation.

More recently, an analysis of resected stage III melanoma in 174 patients evaluated the efficacy of adjuvant anti-PD-1 therapy by mutational subtype. Among the 11 patients with *KIT*-mutant disease, the median disease-free survival was 33 months, compared to 17 months and 9 months in the *BRAF*- and *NRAS*-mutant subgroups, respectively [56]. However, when compared to controls receiving interferon therapy or managed with observation alone, adjuvant PD-1 blockade did not confer a statistically significant survival benefit in the *KIT*-mutant subgroup [56]. These findings raise questions about the relative immunogenicity of *KIT*-driven melanomas in the adjuvant setting, though they still suggest that durable responses to anti-PD-1-therapy are possible.

Central nervous system (CNS) involvement remains a significant clinical challenge in metastatic melanoma. A 2024 retrospective study examined the impact of common somatic mutations, including *KIT*, on the incidence and outcomes of brain metastases during ICI therapy. Among 85 ICI-treated patients, 20 percent developed CNS metastases at some point during treatment [57]. The only patient with a *KIT* mutation developed delayed-onset brain metastases, while no patients with quadruple negative melanoma (lacking mutations in *BRAF*, *NRAS*, *NF1*, or *KIT*) developed CNS involvement. A notable proportion of patients who presented with brain metastases at baseline were able to achieve durable responses and long-term survival following ICI therapy, with many ultimately undergoing elective treatment discontinuation and remaining in remission. In contrast, patients who developed delayed-onset brain metastases universally did so in the setting of systemic progression, often after failure of both ICI and targeted therapy, and this form of CNS progression was uniformly fatal [57]. Previous studies have demonstrated that approximately 25% of patients with *KIT*-mutant melanoma present with a de novo missense mutation in exon 17, many of which are resistant to currently available TKIs [21,55]. McKean et al. observed marked tumor responses to ICI in half of the patients with *KIT* exon 17 mutant melanoma, suggesting that checkpoint blockade may be a viable option for TKI-resistant *KIT* mutations [54].

To better understand the role of ICI in *KIT*-mutant melanoma, combination approaches have also been explored. Compared to TKI monotherapy (ORR 20%), response rates increased to 32% when TKIs were administered concurrently with another agent [54]. While clinical trial data is not available, case reports have documented durable responses to combination therapy [58,59]. For example, one patient with dual *KIT* mutations achieved sustained remission after receiving pembrolizumab plus imatinib following progression on anti-PD-1 monotherapy [58]. Preclinical models support the hypothesis that KIT inhibition may enhance T-cell or NK-cell activity and modulate the tumor microenvironment, though whether this translates clinically remains to be proven [7]. Ongoing clinical trials are investigating combinations of checkpoint inhibitors and targeted therapies in rare melanoma subtypes, including *KIT*-mutant disease [60].

Importantly, *KIT*-mutant melanomas may not be uniformly immunologically “cold,” as some patients have shown responses to PD-1 or CTLA-4 blockade. Current treatment strategies for metastatic *KIT*-mutant melanoma often combine KIT inhibition with immune checkpoint blockade, aiming to leverage both targeted and immune-mediated effects. However, the variability in response, particularly in the adjuvant setting and in CNS progression, highlights the need for prospective studies. Future work should aim to better define the immunogenicity of *KIT*-driven melanomas and clarify the optimal sequencing or combination of ICIs and TKIs to achieve durable clinical benefit.

## 7. Clinical Implications and Future Directions

The most favorable outcomes have been observed in tumors with mutations in exon 11 (e.g., L576P) or exon 13 (e.g., K642E), where agents such as imatinib or nilotinib have yielded an ORR around 21% and a median PFS of 3.7 months [21,22,23,24,29,30,31,32]. Patients must be selected for activating *KIT* mutations to derive benefit, as studies in populations with unselected or amplification-only melanoma have shown limited to no TKI efficacy [61,62].

### 7.1. Clinical Sequencing Guidance

Treatment sequencing in *KIT*-mutant melanoma should be guided by mutation class, therapeutic response patterns, and disease distribution. Patients with activating exon 11 or 13 mutations (e.g., L576P, K642E) generally respond best to first-line TKIs such as imatinib or nilotinib [22,24,27,47]. Among multitarget agents, sunitinib and dasatinib have demonstrated variable efficacy, often constrained by toxicity or short-lived responses, underscoring the need for careful patient selection [33,34,35,36,37].

For patients who progress on TKI therapy or harbor resistant mutations, transition to checkpoint blockade is recommended. Combination approaches incorporating TKIs with ICIs are an emerging strategy that may improve response durability and delay resistance, though evidence is currently limited [54,58,59,60]. In refractory disease, newer generation TKIs such as regorafenib or ripretinib may also offer modest benefit [38,39]. For CNS involvement, ICIs remain preferred due to superior intracranial activity, while avapritinib warrants further study for its potential to penetrate the CNS [54,63].

### 7.2. Critical Unanswered Questions

1. **What are the mechanisms of primary and acquired resistance to TKI?** Mechanisms of resistance in *KIT*-mutant melanomas are not fully understood, but likely involve secondary *KIT* mutations (as in GIST) or activation of downstream pathways like MAPK/PI3K regardless of *KIT* blockade [64]. One study also reported that *KIT*-mutant melanomas tend to exhibit more aggressive histopathologic features, such as ulceration, vascular invasion, and increased Breslow thickness, which may contribute to or reflect intrinsic resistance phenotypes [65]. Most current data, however, are anecdotal or derived from small trials and case reports. Larger, mechanistically focused studies are needed to uncover these mechanisms and suggest second-line treatments or combination approaches.
2. **How to maximize therapeutic efficacy in the management of brain metastases?** Most TKIs have poor penetration in these areas and many *KIT*-mutant patients eventually develop brain metastases [7]. Although certain TKIs have shown anecdotal CNS activity, it is difficult to determine to what extent the response is due to KIT inhibition as opposed to the effects on VEGFR or PDFGR when using multitarget kinase inhibitors. Future strategies could include the development of TKIs with improved blood–brain barrier penetration and the earlier use of systemic therapies in high-risk patients.
  Next-generation TKIs such as avapritinib, which was originally developed for imatinib-resistant GISTs, have shown promising activity in the presence of CNS metastases. In a case of metastatic vulvar melanoma harboring an exon 17 *KIT* mutation, avapritinib produced a favorable CNS response despite prior treatment failure and high tumor burden [63]. A dedicated clinical study evaluating the efficacy of avapritinib in patients with *KIT*-mutant melanoma and CNS metastases would be instrumental in defining its therapeutic role in this setting.
  Beyond pharmacologic optimization, additional strategies to reduce CNS progression may warrant exploration. In other cancers, such as small-cell lung cancer, prophylactic cranial irradiation has been shown to decrease the incidence of brain metastases [66]. While this approach has not been evaluated in melanoma, it remains a theoretical avenue for future investigation.
3. **What is the role of *KIT* amplification alone?** There is controversy over whether *KIT* amplification (without mutation) is a predictive biomarker for therapy response. Many of the clinical trials that currently exist include patients selected for *KIT* mutation or amplification [21,23,24,30]. In GIST, *KIT* over-expression is primarily driven by epigenetic mechanisms and enhancer domains, rather than *KIT* amplification [67]. Biomarker stratification for melanoma treatment based on amplification remains underdeveloped.
4. **How can biomarker-driven clinical trials be developed for *KIT*-mutant tumors?** Few prospective, randomized trials exist that stratify patients by specific *KIT* mutations. Most evidence comes from small phase II trials or retrospective studies, often mixing *KIT*-mutant with *KIT*-amplified or wild-type cases. As a result, there is a need for trials powered to evaluate mutation-specific efficacy (e.g., exon 11 vs. exon 17). In preclinical models of *KIT* K641E-mutant melanoma, a hybrid biomimetic nanovaccine combining tumor and dendritic cell membranes significantly enhanced dendritic cell maturation, T-cell activation, and inhibited tumor growth. These findings suggest that mutation-specific vaccines may offer a promising adjunct to current treatment methods, specifically immunotherapy [68].
5. **What is the biological effect of rare *KIT* mutations?** *KIT* mutations outside of common hotspots (e.g., L576P, K642E) are poorly studied. Rare mutations (e.g., S628N, T632I) show variable drug sensitivity, but lack systematic preclinical or clinical evaluation [20,43]. While common *KIT* mutations appear to be predictive markers for the efficacy of tyrosine kinase inhibitors, there is currently no consensus on how to interpret or treat patients with these non-canonical variants [16]. To better elucidate the biological and therapeutic implications of these rare variants, future efforts should explore murine models incorporating specific rare mutations, alongside continued reporting of clinical cases and inclusion of such mutations in prospective trials.
6. **What is the biological role of KIT signaling in melanocytes of non-hair-bearing acral skin?** KIT is essential in melanocyte development and migration, but its specific function in glabrous (acral) skin melanocytes is poorly defined [6]. Evidence suggests that KIT expression is preserved in these regions and may be uniquely susceptible to oncogenic transformation, but the developmental cues in acral melanocytes remain unclear [69,70].
7. **How are acral melanomas related to acral nevi?** Molecular analyses reveal that while BRAF and NRAS mutations are common in acral nevi, acral melanomas exhibit a broader spectrum of alterations, including frequent *KIT* mutations, structural rearrangements, and copy number variations [70]. These *KIT* mutations are consistently found in acral melanomas but absent in nevi from the same sites, suggesting a distinct oncogenic pathway. These findings raise questions about what triggers KIT-driven transformation in the absence of a nevus precursor.
8. **What is the role of mechanical stress or anatomic factors that contribute to *KIT* mutagenesis in acral melanomas?** Acral skin is subject to chronic mechanical stress, which has been proposed as a contributing factor to the development of acral melanoma through mechanisms of DNA damage [71]. However, whether this physical stress directly promotes *KIT* mutagenesis or preferentially selects for *KIT*-mutant clones remains unclear. A study evaluating the impact of long-term mechanical stress on acral melanoma by comparing clinical and genetic features of lesions on high-pressure areas (heel, forefoot, hallux) versus lower-pressure areas (midfoot, lesser toes) found no significant differences in Breslow thickness or ulceration rates between these sites [72]. Thus, while mechanical stress is broadly implicated in acral melanoma pathogenesis, its specific role in driving *KIT* mutations requires further investigation.

## 8. Conclusions

Significant therapeutic progress has been made in melanoma overall, but *KIT*-mutant disease remains a distinct, challenging subset, defined by unique clinico-pathologic features and KIT pathway activation [1,6,7]. Over the past two decades, understanding of this subtype has grown substantially, particularly regarding its genetic and clinical characteristics. *KIT* mutations tend to occur in acral, mucosal, and CSD melanomas in older patients, and are often mutually exclusive of *BRAF* and *NRAS* mutations [4,7]. The spectrum of mutations spans the *KIT* gene, with hotspots in exons 11 and 13, where L576P and K642E are the most prevalent alterations [7,14,15,16].

Therapeutically, the advent of KIT-targeted tyrosine kinase inhibitors like imatinib provided proof that a subset of these patients can achieve meaningful tumor regressions. Clinical trials have shown ORRs in the range of 16–30% and disease control in 30–70% of *KIT*-mutant melanoma patients on TKIs, especially those with classic activating mutations. However, responses are typically not durable, and resistance, often accompanied by brain metastasis or alternate pathway activation, emerges in many cases, limiting their long-term benefit. Immune checkpoint inhibitors, the standard treatment for melanoma therapy, remain indicated for *KIT*-mutant cases and have demonstrated efficacy in some patients, particularly in the metastatic setting and in TKI-resistant mutations [54,55,56,57,58,59,60]. Immunotherapy has yielded a relatively lower overall response in mucosal and acral tumors; however, their role in the adjuvant setting and in preventing CNS progression remains unclear [57,58,60].

Improving outcomes in *KIT*-mutant melanoma will depend on continued efforts to address several therapeutic challenges. Priorities include developing combination approaches that can prevent or overcome resistance, advancing TKI therapies that are capable of addressing CNS involvement, and continued recruitment of these patients in clinical trials despite their rarity. Additional research into the biology of KIT signaling in melanocytes may reveal new therapeutic vulnerabilities, particularly for rarer *KIT* mutations and amplifications. As the understanding of this melanoma subtype deepens, precision treatment approaches tailored to molecular and clinical features will be essential to improving durable outcomes.

## Figures and Tables

**Table 1:** Summary of clinical trials evaluating KIT inhibitors in advanced melanoma.

Drug (mg)	N	ORR (%)	DCR (%)	mPFS (mo)	mOS (mo)	Mutation Details	Melanoma Subtype	Study
IMA 400 BID	25	16	36	2.8	10.7	Exon 9: 1 (4%) Exon 11: 9 (36%) Exon 13: 6 (24%) Exon 17: 2 (8%) Exon 18: 3 (12%) *KIT* WT: 4 (16%)	Mucosal: 13 (52%) Acral: 8 (32%) CSD: 4 (16%)	Carvajal et al. [21]
IMA 400 QD	78	21.8	60.3	4.2	13.1	Exon 9: 7 (9%) Exon 11: 31 (40%) Exon 13: 16 (21%) Exon 17: 5 (6%) Exon 18: 3 (4%) Multiple mutations: 13 (17%) *KIT* amp: 2 (3%)	Mucosal: 16 (20%) Acral: 42 (54%) CSD: 14 (18%) Others: 6 (8%)	Wei et al. [22]
IMA 400 QD/BID	43	23.3	53.5	3.5	12	Exon 9: 3 (7%) Exon 11: 17 (39%) Exon 13: 9 (21%) Exon 17: 5 (12%) Exon 18: 6 (14%) *KIT* amp: 3 (7%)	Mucosal: 11 (25%) Acral: 21 (49%) CSD: 5 (12%) NSD: 4 (9%) Others: 2 (5%)	Guo et al. [23]
IMA 400 QD/BID	24	29.2	50	3.7 (TTP)	12.5	Exon 11: 9 (38%) Exon 13: 3 (12%) Exon 17: 1 (4%) *KIT* amp: 11 (46%)	Mucosal: 17 (71%) Acral: 6 (25%) CSD: 1 (4%)	Hodi et al. [24]
NIL 400 BID	42	26.2	73.8	4.2	18	Exon 9: 2 (5%) Exon 11: 26 (62%) Exon 13: 13 (31%) Exon 17: 1 (2%)	Mucosal: 20 (48%) Acral: 20 (48%) CSD: 2 (4%)	Guo et al. [27]
NIL 400 BID	26	-	-	3.7	7.7	Exon 9: 0 (0%) Exon 11: 20 (77%) Exon 13: 4 (15%) Exon 17: 2 (8%)	Mucosal: 20 (77%) Acral: 6 (23%) CSD: 0 (0%)	Larkin et al. [28]
NIL 400 BID	25	16	64	6	13.2	Exon 9: 0 (0%) Exon 11: 11 (44%) Exon 13: 8 (32%) Exon 17: 3 (12%) *KIT* WT: 3 (12%)	Mucosal: 10 (40%) Acral: 9 (36%) CSD: 1 (4%) Other: 5 (20%)	Delyon et al. [29]
NIL 400 BID	9	22.2	77.8	2.5	7.7	Exon 9: 0 (0%) Exon 11: 3 (33%) Exon 13: 0 (0%) Exon 17: 0 (0%) Exon 18: 0 (0%) *KIT* amp: 6 (66%)	Mucosal: 1 (11%) Acral: 8 (88%) CSD: 0 (0%)	Cho et al. [30]
NIL 400 BID	19	15.8	52.6	3.3 (TTP)	9.1	Exon 9: 0 (0%) Exon 11: 9 (47%) Exon 13: 5 (26%) Exon 17: 2 (11%) Exon 18: 1 (5%) *KIT* WT: 2 (11%)	Mucosal: 12 (63%) Acral: 4 (21%) CSD: 3 (16%)	Carvajal et al. [31]
NIL 400 BID	42	16.7	57.1	3.3	11.9	Exon 9: 1 (2%) Exon 11: 14 (33%) Exon 13: 4 (10%) Exon 17: 3 (7%) Exon 18: 2 (5%) Exon 11, 13: 2 (5%) Exon 11, 18: 1 (2%) *KIT* WT: 15 (36%)	Mucosal: 12 (29%) Acral: 21 (50%) Cutaneous: 9 (21%)	Lee et al. [32]
DAS 70 BID	22	18.2	50	2.1	7.5	Exon 9: 0 (0%) Exon 11: 14 (63%) Exon 13: 2 (9%) Exon 17: 4 (18%) Exon 11, 13: 1 (5%) Exon 11, 17: 1 (5%)	Mucosal: 8 (36%) Acral: 6 (27%) CSD: 0 (0%) Vulvovaginal: 8 (36%)	Kalinksy et al. [33]
DAS 70 BID	39	5	-	2	13.8	-	Mucosal: 5 (13%) Acral: 5 (13%) Cutaneous: 22 (56%) Ocular: 4 (10%) Unknown: 3 (8%)	Kluger et al. [34]
A: SUN 50 QD, B: SUN 37.5 QD	A: 21, B: 31	44	-	3.1	7.7	Exon 9: 0 (0%) Exon 11: 8 (15%) Exon 13: 2 (4%) Exon 17: 3 (6%) *KIT* WT: 39 (75%)	-	Buchbinder et al. [35]
SUN 50 QD	10	40	50	-	-	Exon 9: 0 (0%) Exon 11: 9 (90%) Exon 13: 1 (10%) Exon 17: 0 (0%) Exon 18: 0 (0%)	Mucosal: 5 (50%) Acral: 3 (30%) CSD: 2 (20%)	Minor et al. [36]
SUN 50 QD	9	22.2	66.2	4.2	26.4	All patients had *KIT* mutations; exact subtypes not detailed	Mucosal: 2 (22%) Cutaneous: 1 (11%) Other: 1 (11%) Non-melanoma: 5 (56%)	Gien et al. [37]
REG 160 QD	23	30.4	73.9	7.1	21.5	Exon 11: 13 (57%) Exon 13: 4 (17%) Exon 17: 4 (17%) Multiple mutations: 2 (9%)	Mucosal: 6 (26%) Acral: 9 (39%) CSD: 3 (13%) Unknown: 5 (22%)	Kim et al. [38]
RIP 150 QD	26	23	-	7.3	-	Exon 11: 8 (31%) Exon 13: 4 (15%) Exon 17: 11 (42%) Exon 18: 1 (4%) Exon 11, 17: 1 (4%) *KIT* amp: 1 (4%)	Mucosal: 15 (58%) Acral: 4 (15%) Desmoplastic: 1 (4%) Splitzoid: 1 (4%) Unknown: 5 (19%)	Janku et al. [39]

Abbreviations: IMA = imatinib; NIL = nilotinib; DAS = dasatinib; SUN = sunitinib; REG = regorafenib; RIP = ripretinib; QD = once daily; BID = twice daily; ORR = overall response rate; DCR = disease control rate; mPFS = median progression-free survival; mOS = median overall survival; WT = wild-type; CSD = chronically sun-damaged; NSD = melanomas on skin without chronic sun-induced damage; N = number of patients; mo = months.

## Data Availability

No new data were created or analyzed in this study. Data sharing is not applicable to this article.

## Abbreviations

The following abbreviations are used in this manuscript:

CSD	Chronically sun-damaged
GIST	Gastrointestinal stromal tumor
TKI	Tyrosine kinase inhibitor
ICI	Immune checkpoint inhibitor
ORR	Objective response rate
DCR	Disease control rate
PFS	Progression-free survival
OS	Overall survival
CR	Complete response
PR	Partial response
CNS	Central nervous system
